# Mercury Hair Concentration among Primary School Children in Malaysia

**DOI:** 10.3390/children4120109

**Published:** 2017-12-14

**Authors:** Nurul Izzah Abdul Samad, Zaleha Md Isa, Rozita Hod

**Affiliations:** 1 Department of Community Health, Faculty of Medicine, Universiti Kebangsaan Malaysia Medical Center (UKMMC), Jalan Yaacob Latif, Bandar Tun Razak, Cheras, 56000 Kuala Lumpur, Malaysia; zms@ppukm.ukm.edu.my (Z.M.I.); rozita.hod@ppukm.ukm.edu.my (R.H.); 2 Environmental and Occupational Health Program, School of Health Sciences, Health Campus, Universiti Sains Malaysia, 16150 Kubang Kerian, Kelantan, Malaysia

**Keywords:** hair mercury, children, fish consumption, mercury poisoning symptoms

## Abstract

The main concern regarding mercury exposure is the adverse health effect on the developing nervous system. The objective of this cross-sectional study was to determine hair mercury levels and their association with socio-demographic characteristics, complaints about mercury poisoning symptoms and the fish consumption pattern among children in Malaysia. A cross-sectional study was conducted among 215 school children aged 11 years old. Hair was collected from the children and the total mercury was analyzed using oxygen combustion–gold amalgamation atomic absorption spectrophotometry. Anthropometric data, a fish consumption questionnaire and mercury poisoning symptoms were collected during a personal interview. The mean hair mercury level among primary school children was 0.63 ± 0.59 µg/g with the geometric mean of 0.47 µg/g. A total of 14% of respondents had hair mercury levels above 1 µg/g. A multiple binary logistic regression analysis outlined that fish consumption of at least one meal per week increased the likelihood of having a high mercury level (odds ratio (OR) 3.7, 95% confidence interval (CI) 1.3–10.4). This study confirms the existence of a mercury burden among Malaysian children and the level is high compared to other regional studies. This study provides important baseline data regarding the mercury level among children in Malaysia.

## 1. Introduction

Mercury is a global pollutant, which comes from natural and anthropogenic sources. Its presence in the environment poses threats and causes concern to the worldwide community. Methylmercury is the most threatening mercuric form to human health as it can alter the nervous system, as well as the cardiovascular system and sensory, visual and auditory functions [1]. Environmental mercury exposure among the general population comes mainly from dietary intake, amalgam filling, and household products such as paint and skin lightening cream [2,3,4]. In recent years, scientists have come to the conclusion that ingestion of one to two meals a week of fish may increase the mercury burden on the body, which will lead to the United States Environmental Protection Agency (USEPA) reference dose (0.1 µg/kg per day) being exceeded [4]. However, the risk of increased mercury levels depends on several criteria, including fish types and their contamination level, as well as frequency of consumption and serving size [5]. With the right number of doses, methylmercury, which has been viewed as a neurotoxicant agent, may have adverse health effects on the fetus and young children.

Children are more susceptible than adults, due to there being a specific mercury exposure pathway to children. Mercury can cross the placental barrier and affect the nervous system of the developing fetus [6,7,8]. Prenatal exposure to low-level mercury can pose a developmental neurotoxicity to children [9]. Aside from prenatal exposure to mercury, children may also be exposed to mercury from their diet and breast milk, or from working or living in an environment associated with mercury exposure and hand–to–mouth activities [1].

Mercury poisoning symptoms can be distinguished into three areas: psychological disturbances; neurological and behavioral disturbances; and body system disturbances. The clinical signs and symptoms of mercury poisoning vary among individuals who are exposed to the metal, and it usually depends on the dose, the length of exposure, and the route of exposure [10]. Acute toxicity is usually associated with the inhalation of elemental mercury or the ingestion of inorganic mercury, whereas chronic toxicity is more common to the exposure to methylmercury. The common symptoms of chronic toxicity include reduced cognitive functions and memory disturbances, such as poor concentration, fatigue, and irritability [11].

Early screening of the mercury level in an individual, especially in young children, is crucial so that we can predict the extent of exposure someone is experiencing [12]. Hair mercury testing is suitable for use since the method is non-invasive and the mercury content in hair correlates well with dietary methylmercury intake [9]. Furthermore, the total mercury in hair represents almost 80–95% of methylmercury concentrations in the blood, thus making hair sample analysis accepted as a dependable method to estimate the dose. Countless studies of mercury exposure were performed on the adult population; however, no data has been reported on the level of mercury among children in this country. This paper aims to explore hair mercury levels and to predict factors associated with high mercury levels among Malaysian children.

## 2. Materials and Methods

### 2.1. Study Population and Sampling

This study adopted a cross-sectional study design, whereby a simple random sampling was used to recruit children aged 11 years old from primary schools in Negeri Sembilan, Malaysia. The school administrator provided the students’ name list. Eligibility criteria required the subjects to be year 5 students, to have Malaysian citizenship, and to have received written consent from their parents or guardian. The consent was obtained prior to the data collection. Meanwhile, the exclusion criteria for the study included students who were absent on sampling day, who did not understand the Malay language, and students who were bald or had less hair. The respondents were interviewed and the data collected was comprised of residential information, health status (including mercury poisoning symptoms) and the number of dental amalgam fillings (if any), height and weight for a body mass index (BMI-for age) calculation, as well as the frequency of fish consumption using a Food Frequency Questionnaire (FFQ). BMI-for age was used as a screening tool to assess risk of being overweight among children and adolescents. Their BMI was compared against age, based on their gender. Their mothers were also interviewed using a self-administered questionnaire regarding their pregnancy and breastfeeding history, their children’s parity, and their residential information. A question on their maternal diet during pregnancy was asked, and whether they were a frequent fish-eater or a non-frequent fish-eater. Frequent fish-eaters were defined as mothers who normally consumed fish at least once a week during their pregnancy, while non-frequent fish-eaters were defined as consuming less than one fish meal per week. The final study samples included 215 children with accessible hair mercury samples and completed questionnaires. All of the procedures were executed after we received an ethical approval from the Research and Ethical Committee at the University Kebangsaan Malaysia Medical Center (FF–2016–393).

### 2.2. Biological Sample Analysis

Hair samples of approximately 3 cm in length were cut from the root of the occipital region of the head. The minimum amount of hair required per respondent was around 100 to 150 strands, and the hair samples were kept individually in a sealed and labeled envelope. All of the samples were sent to the National Institute for Minamata Disease (NIMD) in Japan for total mercury analysis. The analysis was carried out by following the oxygen combustion–gold amalgamation method using the mercury analyzer (MA-2000, Nippon Instruments Corporation: Takatsuki, Japan) without any pre-treatment procedure. The limit of detection was 0.17 ng/g. Meanwhile, the reference limit established by USEPA for hair mercury was 1 µg/g, corresponding to a reference dose of no greater than 0.1 µg/kg bw/day.

### 2.3. Statistical Analysis

All of the variables were tested for normal distribution using the Kolmogorov–Smirnov test. Student’s *t*-test and one-way analysis of variance (ANOVA) were used to measure the statistical differences in hair mercury concentrations by gender, location, race, fish consumption, smoking status, and an amalgam filling. Analysis of covariance (ANCOVA) was further tested to examine the effect of smoking status and of an amalgam filling on the mercury concentrations controlling for fish consumption. Since the total hair mercury levels were not normally distributed, they were normalized by log_10_ transformation before the analyses. A multiple logistic regression analysis was performed to determine factors associated with higher mercury levels, controlling for gender. Fish consumption was measured under the categories of not eating fish at all (never), eating one fish meal per month (monthly), eating one fish meal per week (weekly), and eating one fish meal every day (daily). For the multiple logistic regression analysis, fish consumption was categorized as having one fish meal or more per week or having less than one meal per week. Statistical significance was defined as *p* < 0.05. Statistical analysis were conducted using IBM SPSS Statistics for Windows, Version 21.0 (IBM Corp.: Armonk, NY, USA).

## 3. Results

The geometric mean value for hair mercury levels and the general characteristics of respondents in this study are presented in Table 1. The arithmetic mean hair mercury concentration was 0.63 µg/g and its geometric mean was 0.47 µg/g. This study comprised of 44.2% male respondents, who had a mean mercury level slightly lower than that of the female respondents; however, it was not significantly different. A total of 57.7% of the children lived in a rural area, which is located near the coast which has oil refineries nearby. Children who lived in the rural area had a significantly higher mean mercury level compared to that of the urban children (*p* < 0.05). Other than that, 62.3% of the respondents ate fish regularly, which is reflected in the higher mercury burdens on their bodies, compared to those who consumed less fish. Mercury concentration also elevated according to the respondents’ frequency of fish consumption (*p* = 0.047). Children who had an amalgam filling were shown to have a higher mercury level compared to those who did not have an amalgam filling (*p* < 0.05). Meanwhile, children who were passive smokers did show a higher mercury concentration compared to those who were non-smokers; however, the difference was not statistically significant (*p* = 0.072). Further analysis of covariance showed that there were no significant differences in mercury concentration with smoking status [F(1, 212) = 2.66 *p* = 0.103] and with an amalgam filling [F(1, 212) = 2.41 *p* = 0.121], after controlling fish consumption.

**Figure 1:**
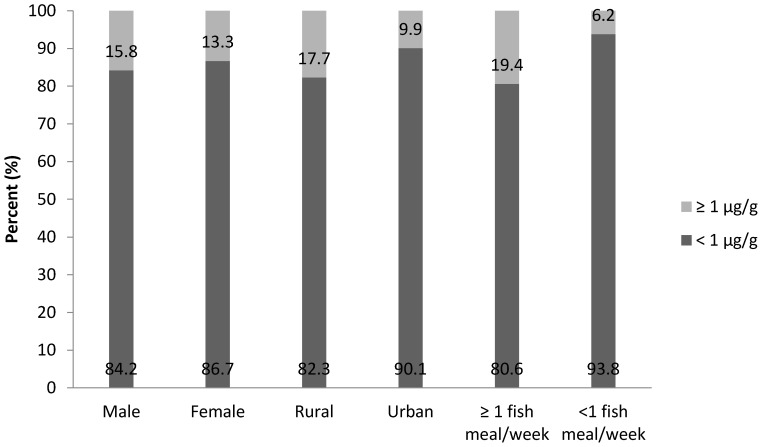
Mercury distribution among respondents according to gender, location and frequency of fish consumption of the study.

Figure 1 presents mercury distribution according to children’s gender, location and frequency of fish consumption. This study presented that 15.8% of male and 13.3% of female respondents had a mercury level above the reference dose suggested by the USEPA for the general population. Meanwhile, 17.7% of respondents who lived in the rural area had a mercury level above the reference limit, while 9.9% of those who lived in the urban area belong in the same category. Respondents who had at least one fish meal per week presented 19.4% of those who had a mercury level above 1 µg/g.

**Figure 2:**
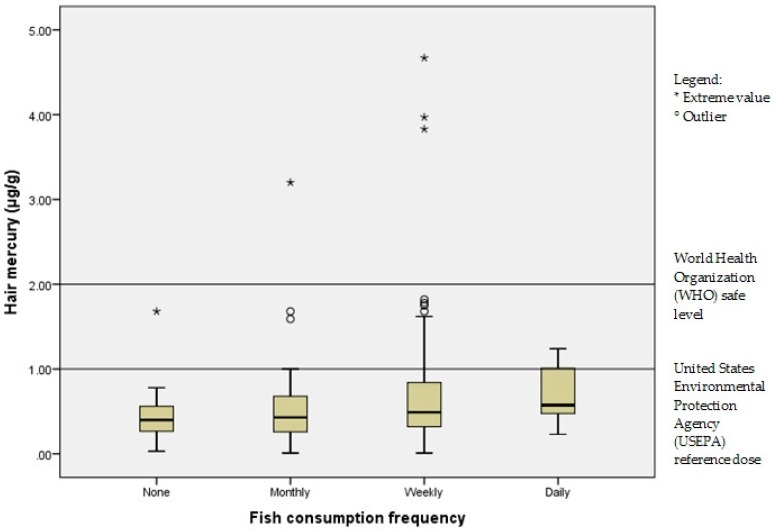
Distribution of hair mercury according to fish consumption frequency.

Figure 2 depicts the distribution of hair mercury according to the frequency of fish consumption by the respondents. A total of 14.4% of the respondents exceeded the USEPA reference dose (1 µg/g), while 1.86% of the respondents (*n* = 4) exceeded the World Health Organization (WHO) safe level of 2 µg/g [12]. A one-way ANOVA was conducted to compare the distribution of hair mercury level with the frequency of fish consumption, under the categories of no consumption, monthly, weekly or daily consumption. There was a statistically significant effect of fish consumption frequency on hair mercury levels at the *p* < 0.05 level for the four conditions [F(3, 211) = 2.69, *p* = 0.047]. Post-hoc comparison using the Tukey’s honest significant difference (HSD) method indicated that the comparison did not differ significantly between the different groups (*p* > 0.05).

Table 2 shows the comparison of total mercury levels among Malaysian children with other countries. Hair mercury concentration may differ based on the geographic location of a country and the criteria for the sampled population, such as their lifestyle and dietary intake. The mercury levels among Malaysian children were comparable to children who lived in Korea and were slightly higher than in children of Germany, China, the United States, and Belgium. High-risk populations such as the one that lived near the river bank in Brazil and high fish consumers such as Japanese and Spanish children, did show higher mercury levels compared to those of Malaysian children.

Table 3 depicts the results of a multivariate logistic regression analysis showing the factors associated with a higher mercury level. The likelihood of having a higher mercury level is influenced by the total amount of fish consumption per month (odds ratio (OR) 3.69, 95% confidence interval (CI) 1.31–10.36). Other factors did not show any significant association (*p* > 0.05); however, certain trends can be observed. Respondents who lived in a rural area were 1.50 times more likely to have a higher mercury level and for every 10 kg/m^2^ increased of BMI for-age, the likelihood to have a higher mercury level increases by 10 times. A mother who regularly ate fish during her pregnancy was 1.92 times more likely to have a higher mercury level.

## 4. Discussion

The children in this study had a relatively higher mean of mercury levels compared to that of children in the Czech Republic (mean 0.18 µg/g) and children in the United States (mean 0.30–0.40 µg/g) [4,23]. It was found that the children in this study had a comparable mean mercury concentration with the children in other higher income countries, such as Korea and had a higher mean compared to China [15,18]. This result could probably be due to a higher frequency of fish consumption among those respondents. Interestingly, mean mercury levels increased in parallel with the frequency of fish consumption as observed in this study. This current study showed that almost 62.3% of the children consumed fish in at least one meal per week, whereas 24.8% of children in the National Health and Nutrition Examination Survey (NHANES) study consumed fish three or more times in the past 30 days [16].

Meanwhile, in another study, fish consumption frequency was in agreement with the higher mercury burden, where children who ate fish once a week or more had an increased mercury level in their bodies [4]. Mediterranean children also shared the same pattern, where children who ate fish for at least two meals per week had the highest hair mercury concentrations [22]. The Japanese population is known as the world’s largest fish consumers, thus, it is no doubt that their population had a higher mean mercury level [20]. Malaysia has been considered among the highest fish consumers of Asian countries; the country falls right behind Japan and is the biggest fish consumer in Southeast Asia [24]. Therefore, studies among Malaysian adult communities also noted the same trend. The studies showed that besides consumption frequency, the amount of fish consumed and type of fish consumed contributed to a significant correlation with total hair mercury [25,26,27,28,29]. For other Southeast Asian countries such as Singapore, Thailand, Indonesia, and Cambodia, elevated mercury levels were anticipated, due to the fact that the mercury level in fish consumed by the communities varies according to the species. The fish consumption rate also differs compared to that of Malaysian people [26,30,31,32].

The result of this study indicated that children who lived in a rural area had a significantly higher mean mercury concentration compared to that of children who lived in an urban area. The difference might be due to their frequency of fish intake. In total, 63.4% of the rural children ate fish for at least one meal per week, whereas around 36.6% of urban children showed the same dietary pattern (*p* < 0.05). Children who consumed fish daily had almost twice the mercury burden in their body, compared with children who did not consume fish. The findings of the current study are consistent with Italian residents who lived near a coastal area and had chlor-alkali plants nearby, who noted the same pattern [33].

Meanwhile, a total of 72.6% of mothers in the rural area consumed fish heavily during their pregnancy, compared to 60.4% of urban mothers who did so (*p* = 0.06). On the other hand, children who had an amalgam filling had a significantly higher level of mercury, compared to those who had no amalgam filling (*p* = 0.033). Amalgam filling was known to be one of the routes of entry for mercury vapor into the body and it had previously been observed as causing the increment of mercury in the body [15,34].

It is interesting to note that this study was the first study to date that reported the mean mercury levels among Malaysian children. Previous studies were more focused on the adult population, those who were exposed to mercury environmentally, and those who lived in fishing villages [25,27,35,36]. Surprisingly, the previous findings revealed that the majority of the adult population in Malaysia had mercury levels exceeding the USEPA reference dose. Hair mercury concentrations were known to increase with age, thus, resulting in a high level of mercury in the adult population compared to that of children [14]. This factor, however, is still debatable because of other factors such as dietary patterns and amalgam fillings which also play a role in the mercury burden in the body [23,37].

Further binary logistic regression analysis showed that total fish consumption was positively associated with the likelihood of having higher mercury levels. This study concluded that children who ate fish for at least one meal per week were over four times as likely to have a high level (≥1 µg/g) of mercury, compared to children who ate fish less than three times per month. Previous studies confirmed that children who ate fish several times per week had a higher mean mercury level compared to those who ate less [4,9,14]. Children’s BMI, the area of residency, and mother’s diet during pregnancy were shown to correlate with a trend towards a high mercury level, but were not statistically significant (*p* > 0.05). The previous study also noted the same pattern, where mercury level may be affected by BMI and location of residency [15,38]. BMI is one of the biological factors that may influence metabolism and concentrations of heavy metals in the body. Previous studies did suggest that fish-eating during pregnancy significantly contributed to a moderate level of mercury in infants and children, which resulted in a detrimental effect on neurodevelopment [39,40,41,42,43,44]. Nevertheless, some studies found no association [45,46,47]. However, adjustment for the maternal fish intake was based on mother’s recall, thus did not enunciate any nutrient assessment. A moderate consumption of low mercury-contaminated fish is recommended as fish are known to considerably benefit children’s intelligence development. Moreover, studies have shown that oily fish—which contain polyunsaturated fatty acids, especially omega-3 fatty acid—have a beneficial intelligence effect, while lean fish, which usually contain a higher mercury level, evoked an adverse intelligence effect [48,49,50]. On the other hand, local studies suggested that predatory fish from the demersal zone and higher marine trophic level induced a greater mercury burden [5,29]. This is to be expected since bioaccumulation of mercury concentrations in fish would have happened at higher trophic levels [51,52,53].

Mercury levels might increase as children grow older since they will still be exposed to a seafood diet and other environmental factors. Regardless of the existence of a relationship between fish consumption and mercury, this study did not expect the communities, especially the children, to restrict their fish-eating diet. The public should be aware of the mercury contamination in fish. The risks of mercury exposure through diet should not outweigh the benefits that fish can provide. Special attention should be given to female children with mercury levels exceeding the reference dose as they will become mothers one day and may transmit the mercury to their unborn offspring.

## 5. Conclusions

This study has shown that 14.4% of children exceeded the reference limit set by the USEPA. The mean hair mercury level (0.63 ± 0.59 µg/g) did not differ from other countries with similar geographic characteristics. The findings enhanced our understanding of mercury levels among children in Malaysia. This result will help future studies on mercury exposure and it provides important baseline data regarding the mercury levels of Malaysian children.

## Figures and Tables

**Table 1:** Hair mercury levels and general characteristics of the respondents (*N* = 215).

Characteristics	*N* (%)	Total Hg, µg/g GM (Range) ^a^	*p*-Value
Total	215 (100)	0.47 (0.01–4.67)	
Gender			
Male	95 (44)	0.46 (0.01–4.67)	0.867
Female	120 (56)	0.47 (0.03–3.97)	
Location			
Urban	91 (42)	0.41 (0.01–3.97)	0.001 *
Rural	124 (58)	0.55 (0.03–4.67)	
Race			
Bumiputera	205 (95)	0.47 (0.01–4.67)	0.458
Non-Bumiputera	10 (5)	0.57 (0.12–3.97)	
Fish consumption			
Never	11 (5)	0.33 (0.03–1.68)	0.047 *^,+^
Monthly	70 (33)	0.40 (0.01–3.20)	
Weekly	122 (57)	0.51 (0.01–4.67)	
Daily	12 (6)	0.64 (0.23–1.24)	
Smoking status			
Non-smoker	121 (56)	0.43 (0.01–1.82)	0.072
Passive smoker	94 (44)	0.53 (0.09–4.67)	
Amalgam filling			
No	162 (75)	0.44 (0.01–4.67)	0.033 *
Yes	53 (25)	0.55 (0.14–1.82)	

* Significant at *p* < 0.05; ^a^ Geometric mean; *p*-value for *t*-test except; ^+^ one-way analysis of variance (ANOVA).

**Table 2:** Comparison of total mercury in hair (µg/g) among Malaysian children and other countries.

Location	AM ^a^ (µg/g)	GM ^b^ (µg/g)	Range	*N*	Remarks
Malaysia (this study)	0.63	0.47	0.01–4.67	215	11 years old
Tarragona, Spain [13]	-	0.77	0.30–2.44	233	6–16 years old
Belgium [14]	-	0.20	<LOQ ^d^–1.99	129	6–11 years old
China [15]	0.11	0.10	0.03–0.52	184	10–13 years old
United States [16]	0.22	0.12	0.18–0.25	838	1–5 years old
German [17]	0.23	0.18	0.06–1.70	245	8–10 years old
Korea [18]	0.74	0.62	0.12–3.46	112	<15 years old
Korea [15]	0.76	0.69	0.27–2.24	54	10–12 years old
Spain [19]	0.94	-	0.19–5.63	136	Pre school
Japan [20]	1.65 ^c^	-	0.27–6.32	327	7 years old
Maranhao, Brazil [21]	2.27	-	0.13–9.54	139	<12 years old
Spain [22]	1.4	0.99	0.04–10.0	302	4 years old

^a^ Arithmetic Mean; ^b^ Geometric Mean; ^c^ Median; ^d^ Limit of quantification.

**Table 3:** Factors attributed to high mercury level.

Variable	B	S.E.	Odds Ratio (95% CI)	*p* Value
Location (rural)	0.40	0.44	1.50 (0.63–3.55)	0.359
BMI for age	0.05	0.04	1.06 (0.98–1.14)	0.169
Fish consumption once a week	1.31	0.53	3.69 (1.31–10.36)	0.013 *
Mom ate fish during pregnancy	0.65	0.49	1.92 (0.73–5.02)	0.186

* Significant at *p* < 0.05; BMI: body mass index; B: Coefficient; S.E.: Standard error; CI: Confidence interval.
